# *Mycoplasma pneumoniae* infection with neurologic complications

**DOI:** 10.1186/1824-7288-41-S2-A67

**Published:** 2015-09-30

**Authors:** Alberto Spalice

**Affiliations:** 1 UOC Neurologia Pediatrica, Sapienza Università Roma, Viale Regina Elena 324, Rome, Italy

## 

*Mycoplasma pneumoniae* (*M. pneumonia*) is one of the important causes of upper and/or lower respiratory tract infections during childhood. Central nervous system (CNS) related findings and complications are most commonly seen and have been described in patients with *M. pneumoniae* infections [1,2]. Patients suffering *M. pneumoniae* infection may have varying degrees of neurological complications at a ratio of approximately 6-7% [1,2]. Neurological manifestations include encephalitis, transverse myelitis, acute disseminated encephalomyelitis (ADEM), Guillain-Barre syndrome, and thromboembolic stroke [2]. The time period between the onset of respiratory symptoms and neurological symptoms varies 2 to 14 days [3,4]. More than 80% of patients with CNS findings have concomitant respiratory infection [5].

Central nervous system complications have been reported with Mycoplasma infection. Cerebellar syndrome, polyradiculitis, cranial nerve palsies, aseptic meningitis, meningoencephalitis, acute disseminated encephalomyelitis, coma, optic neuritis, diplopia, mental confusion and, acute psychosis secondary to encephalitis, cranial nerve palsy, brachial plexus neuropathy, ataxia, choreoathetosis, and ascending paralysis (Guillain-Barre syndrome) are neurologic complications seen with *M. pneumoniae* infection [1].

Neuroimaging may reveal normal findings or focal diffuse edema in cases of encephalitis or meningoencephalitis. Patchy asymmetric or diffuse signal change of gray and white matter may be seen in patients with ADEM with multifocal, asymmetric foci of high signal intensity on FLAIR and T2 weighted images. A focal infarction may be seen with *M. pneumoniae* related stroke [4].

Treatment of neurologic complications of *M. pneumoniae* is controversial. Treatment may be adjusted according to infection mechanism such as antibiotics, corticosteroids, intravenous immunoglobulin [3-5]. Antimicrobial treatment, especially macrolides, may be sufficient for CNS involvement associated with *M. pneumoniae*, beside the beneficial effect of treatment with steroids this treatment must be considered with direct invasion of CNS by the organism when other causative agents have been excluded. Plasma exchange has also been reported and seemed to be beneficial.
